# Erratum: Seepage from an arctic shallow marine gas hydrate reservoir is insensitive to momentary ocean warming

**DOI:** 10.1038/ncomms16126

**Published:** 2017-06-30

**Authors:** Wei-Li Hong, Marta E Torres, JoLynn Carroll, Antoine Crémière, Giuliana Panieri, Haoyi Yao, Pavel Serov

Nature Communications 8: Article number:15745 ; DOI: 10.1038/ncomms15745 (2017) Published: 06 07 2017; Updated: 06 30 2017

The financial support for this Article was not fully acknowledged. The Acknowledgements should have included the following:

The publication charges for this article have been funded by a grant from the publication fund of UiT The Arctic University of Norway.

