# Supplementary material for: Seepage from an arctic shallow marine gas hydrate reservoir is insensitive to momentary ocean warming
Source: Nat Commun. 2017 Jun 7;8:15745. doi: 10.1038/ncomms15745 (PMC5477557; doi:10.1038/ncomms15745)
Supplement: Supplementary Information — Supplementary Figures, Supplementary Tables and Supplementary References [file ncomms15745-s1.pdf]

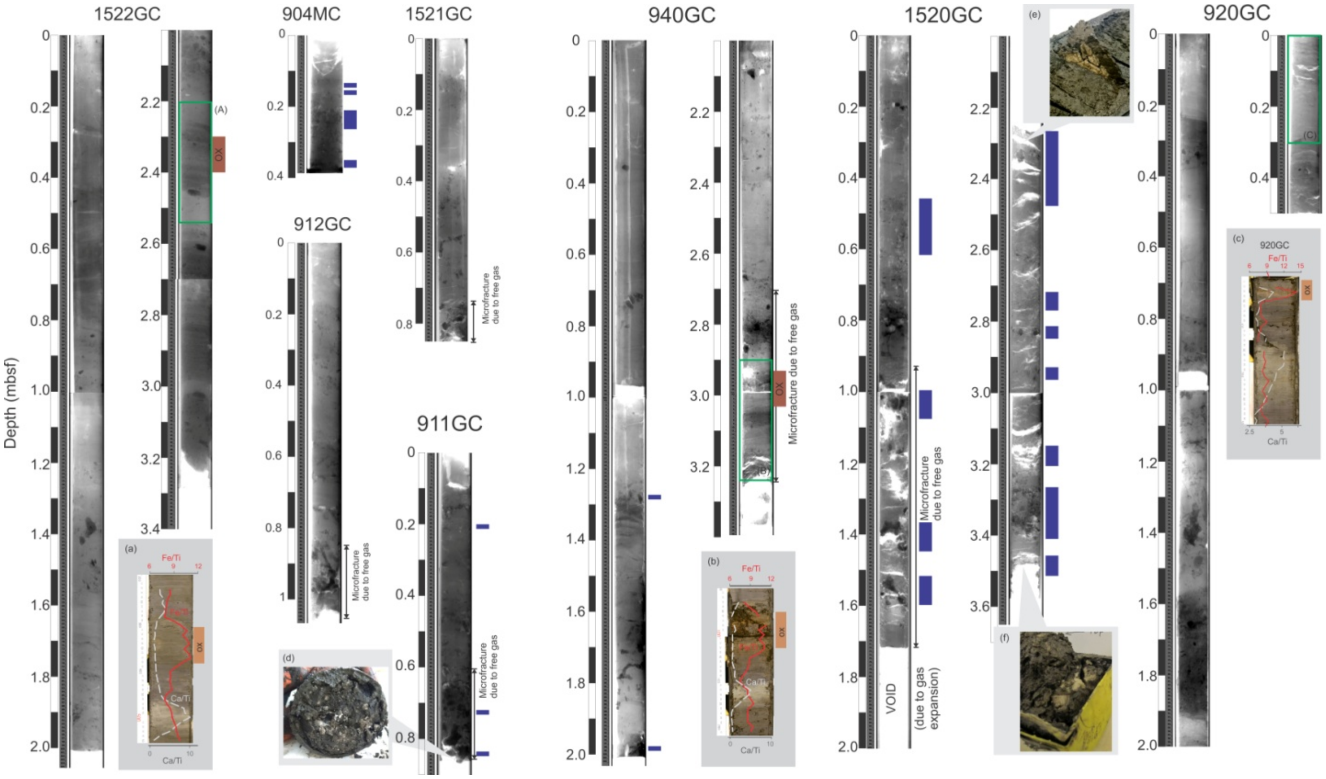

Supplementary Figure 1: Sediment cores from Storfjordrenna GHMs X-ray images of the investigated sediment cores. Purple bars show approximated depths where authigenic carbonates were recovered. Zones where microfractures were observed are demarked by vertical arrows. The presence of oxidized zones in the cores, clearly identified by a color change and an increase in Fe/Ti ratios in the XRF data, are shown in insets a-c. Images showing gas hydrates are also included (insets d-f), showing the location in the cores where they were found.

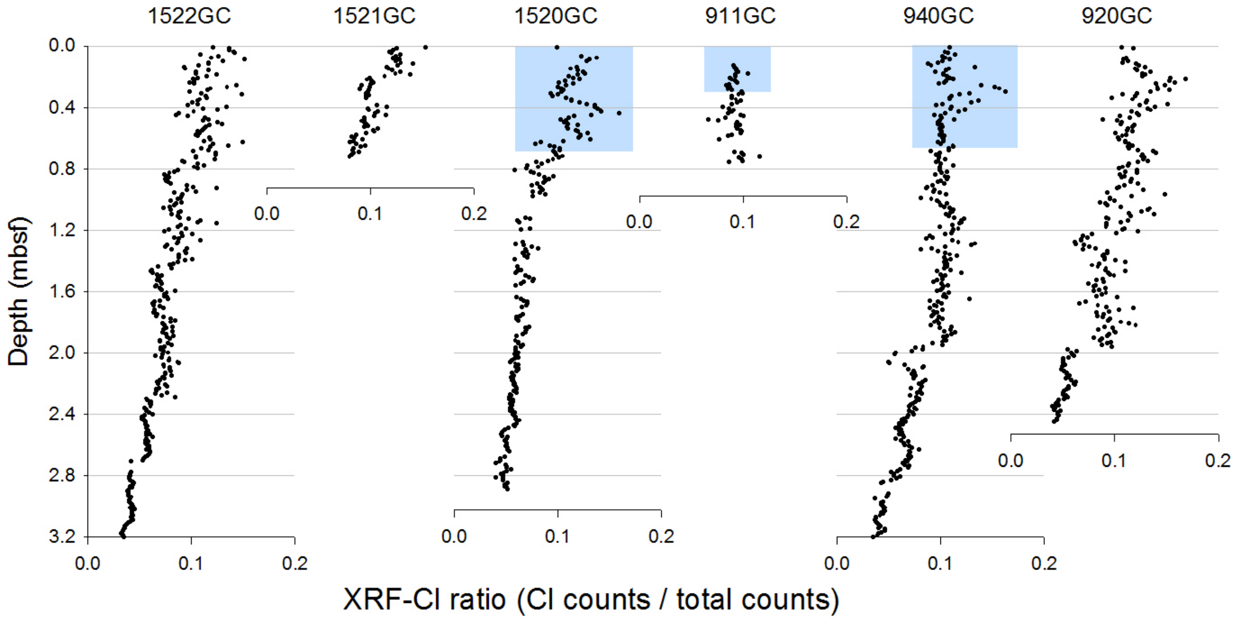

Supplementary Figure 2: Cl ratio of the selected sites. Cl ratio was calculated by divided the raw Cl counts from XRF scanning with the summation of counts from various major elements (Al, Si, S, Cl, K, Ca ,Ti, Fe, Mn, and Rh). Blue areas mark the sediment depths above the kinks in porewater profiles (i.e., near-seawater composition). In general, the Cl ratio decreases with depth indicating reduction in porosity due to sediment compaction. We observed no difference within the sediment depths above the kinks compared to deeper sediments or from other sites.

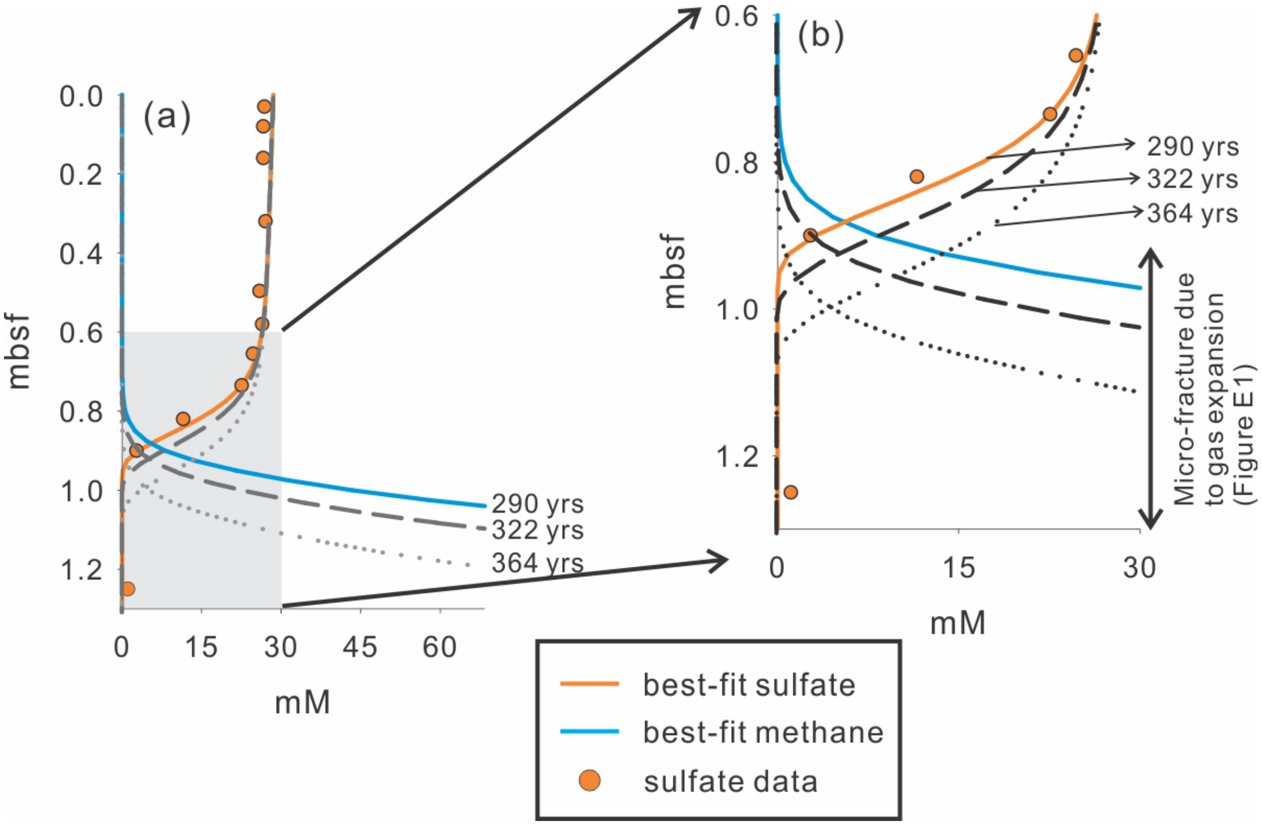

Supplementary Figure 3: Sensitivity tests of the 1-D simulation (reduced model) of the porewater profiles We performed a test using data from 1520GC, the site with the highest porewater sampling resolution, by assigning progressively weaker methane supplies to test the sensitivity of the model to our time estimates. A weaker methane supply will shoal the SMTZ more slowly and therefore result in a larger time estimate for seepage. A longer time span will allow diffusion to reduce the gradient contrasts between the two segments of the profiles. A weaker methane supply will also saturate the porewater at greater depth resulting in the appearance of gas hydrate and/or gas fractures deeper than where they were observed. In the best fit scenario, it takes 290 years to fit the measured data (with tortuosity equals to 1.5). If the methane flux at depth is slightly decreased, it takes 322 years to fit the observed sulfate profile although the predicted depth for gas hydrate saturation is 25cm deeper than the depth where free gas expansion was observed.

Supplementary Table 1: Mineralogy and carbon isotopic signature of the authigenic carbonate nodules.

Depth (cmbsf)	% Mg-calcite	$\delta^{13}C$ (‰ VPDB)	Depth (cmbsf)	% Mg-calcite	$\delta^{13}C$ (‰ VPDB)
911GC			***1520GC***		
20	15	-25.4	46.5	NA	-27.32
72	15	-29.8	51.5	NA	-26.21
83	17	-30.1	56.5	NA	-26.94
904MC			61.5	NA	-28.39
13	16	-29.91	103.5	NA	-30.59
16	16	-30.79	107.5	NA	-28.71
21	16	-29.46	108	16	-30.61
23	16	-29.46	143	15	-29.29
26	14	-28.99	157.5	NA	-24.84
39	16	-27.74	159	15	-29.85
40	15	-27.38	162.5	NA	-26.89
940GC			227.5	NA	-27.13
128	15	-31.97	232.5	NA	-27.93
199	17	-35.40	247.5	NA	-23.58
1521GC			272.5	NA	-23.16
36	16	-30.68	277.5	NA	-22.63
47	15	-27.38	282.5	NA	-26.47
50	14	-28.53	297.5	NA	-24.67
57.5	15	-29.43	317.5	NA	-23.28
65	14	-27.95	322.5	NA	-25.54
			327.5	NA	-25.69
			328.5	16	-26.66
			332.5	NA	-26.39
			337.5	NA	-27.14
			342.5	NA	-27.51
			347.5	NA	-24.66

NA: not available

Supplementary Table 2: ^{14}C dating results of planktonic foraminifera from 1522GC

Sample label	Lab #	Depth	Radiocarbon age (yrBP)	2-Sigma calibrated age (cal yrBP)	$\delta^{13}C$ (‰VPDB)
CAGE 15-6HH 1522GC 70-72	Beta-456617	70-72	8690 ± 40	9405-9105	-0.35
CAGE 15-6HH 1522GC 220-222	Beta-456618	220-222	13820 ± 60	16210-15835	-0.10

Supplementary Table 3: Temperature data selecting criteria

Water depth (mbsl)	Longitude range (^{o})	# of data	Average BWT (^oC)	BWT standard deviation (^oC)	Max BWT (^oC)	Min BWT (^oC)
200-500	12-20	2318	1.80	1.49	6.92	-1.93
200-500	15-17*	899	1.85	1.38	5.58	-1.89
300-450	12-20	933	1.77	1.34	6.92	-1.76
300-450	15-17	414	1.44	1.41	4.65	-1.76

* Included Storfjordrenna GHMs

More than two thousand temperature measurements were collected from the World Ocean Database (WOD)[1] that cover 200-500 meters water depth and are within an 8° by 2° area surrounding the GHMs (75 to 77 ^{o}N and 12 to 20 ^{o}E). These measurements occurred from 1871 until 2009, mostly during spring to late fall with a few measurements from winter months. We also include five temperature measurements which were taken during our two 2015 cruises. The average bottom water temperature does not differ much (from 1.85 to 1.44 ^{o}C) with different geographic coverage and water depth but the maximum temperatures show slightly higher variation.

Supplementary Table 4: Porewater and solid species considered in the model

Primary species	$H_{2(aq)}$	$CH_{4(aq)}$	HS^-	SO_4^{2-}	HCO_3^-
	Cl^-	Na^+	Ca^{2+}	Mg^{2+}	NH_4^+
	Fe^{2+}	H^+			
Secondary species	$CO_{2(aq)}$	CO_3^{2-}	$NH_{3(aq)}$	$H_2S_{(aq)}$	S^{2-}
Gases	$CO_{2(g)}$	$H_2S_{(g)}$	$CH_{4(g)}$		
Minerals	Calcite-Ca	Calcite-Mg	$(CH_2O)_1(NH_3)_{0.169}$	Pyrite	$Fe(OH)_3$
	Goethite				

Supplementary Table 5: Reactions and corresponding Gibbs free energy of reaction considered in the comprehensive model

(a) Homogeneous reactions			*ΔG_{rxn}*
Acid-base	$H_2O+CO_2 \rightarrow HCO_3^-+H^+$		
	$HCO_3^- \rightarrow CO_3^{2-}+H^+$		
	$NH_4^+ \rightarrow NH_{3(aq)}+H^+$		
	$H_3PO_4 \rightarrow H_2PO_4^-$		
	$H_2PO_4^- \rightarrow HPO_4^{2-}$		
	$HPO_4^{2-} \rightarrow PO_4^{3-}$		
	$H_2S_{(aq)} \rightarrow HS^-+H^+$		
	$HS^- \rightarrow S^{2-}+H^+$		
Gas-dissolvent	$CH_{4(g)} \rightarrow CH_{4(aq)}$		
	$CO_{2(g)} \rightarrow CO_{2(aq)}$		
	$H_2S_{(g)} \rightarrow H_2S_{(aq)}$		
Fermentation	ferm: $0.042C_6H_{12}O_6 + 0.167H_2O \rightarrow$ $0.083CH_3COO- + 0.167H_2(aq) + 0.167H^+ + 0.083HCO_3^-$	(S1)	0.715
Aquatic redox	hySR: $\frac{1}{2}H_{2(aq)} + \frac{1}{8} SO_4^{2-} + \frac{1}{8}H^+ \rightarrow \frac{1}{8}HS^- + \frac{1}{2}H_2O$	(S2)	-32.896
	acSR: $\frac{1}{8}CH_3COO^- + \frac{1}{8}SO_4^{2-} \rightarrow \frac{1}{8}HS^- + \frac{1}{4}HCO_3^-$	(S3)	-5.944
	AOM: $\frac{1}{8}CH_{4(aq)} + \frac{3}{8}H_2O \rightarrow \frac{1}{2}H_{2(aq)} + \frac{1}{8}HCO_3^- + \frac{1}{8}H^+$	(S4)	28.676
	hyME: $\frac{1}{2}H_{2(aq)} + \frac{1}{8}HCO_3^- + \frac{1}{8}H^+ \rightarrow \frac{1}{8}CH_{4(aq)} + \frac{3}{8}H_2O$	(S5)	-28.676
	acME: $\frac{1}{8}CH_3COO^- + \frac{1}{8}H_2O \rightarrow \frac{1}{8}CH_{4(aq)} + \frac{1}{8}HCO_3^-$	(S6)	-1.826

Supplementary Table 5 (cont.)

(b) Heterogeneous reactions			
Calcite-Ca&Mg	$(Ca ,Mg) CO_{3(s)} + H^+ \rightarrow (Mg^{2+} ,Ca^{2+}) + HCO_3^-$	(S7)	-11.54
OM-hydrolysis	$(CH_2O)_1(NH_3)_{0.169} + 0.169H^+ \rightarrow 0.167C_6H_{12}O_6 + 0.169NH_4^+$	(S8)	
Pyrite	$FeS_{2(s)} + H_{2(aq)} \rightarrow Fe^{2+} + H_2S_{(aq)}$	(S9)	87.860
Goethite dissolution	$FeOOH_{(s)} + 2H^+ + ½H_{2(aq)} \rightarrow Fe^{2+} + 2H_2O$	(S10)	-73.49
H_2-iron reduction	$Fe(OH)_{3(s)} + H^+ + H_{2(aq)} \rightarrow Fe^{2+} + H_2O$	(S11)	-73.345

Supplementary Table 6: Kinetic constants for all reactions

	S1	S2	S3	S4	S5	unit
$CaCO_3$	1E-6.8	1E-6.8	1E-6.8	1E-5.3	1E-5.3	Mol m^-2 sec^-1
$MgCO_3$	1E-8.5	1E-8.5	1E-8.5	1E-6.8	1E-6.8	
OM	1E-8.9	1E-8.9	1E-8.9	1E-8.9	1E-10	
$Fe(OH)_3$	1E-5.4	1E-5.4	1E-5.4	1E-6	1E-6	
Pyrite	1E-18.5	1E-18.5	1E-18.5	1E-18.2	1E-18.2	
Goethite	1E-8	1E-8	1E-8	1E-5	1E-5	
CH_4 input	1E-6.8	1E-6.8	1E-7.5	1E-3	1E-3	
$R_{max\text{-}ferm}$			1.29			Mol L^-1 yr^-1
$R_{max\text{-}hySR}$			5			
$R_{max\text{-}acSR}$			1.15			
$R_{max\text{-}AOM}$			50			
$R_{max\text{-}hyME}$			2.22			
$R_{max\text{-}acME}$			1.12			
$K_{glaucose\text{-}ferm}$			1E-3			M
$K_{H2\text{-}hySR}$			1E-8			
$K_{acetate\text{-}acSR}$			1E-4			
$K_{CH4\text{-}AOM}$			1.5E-3			
$K_{H2\text{-}hyME}$			1E-6			
$K_{acetate\text{-}acME}$			5E-3			
$I_{Fe\text{-}hySR}$			4E-7			M
$I_{Fe\text{-}acSR}$			4E-7			
$I_{SO4\text{-}hyME}$			1E-4			
$I_{SO4\text{-}acME}$			1E-4			
time	0.25	0.5	900	2.5	0.7	yr
Δt	Fluid: 0.001/0.05<br>Sed: 0.01/0.05	0.025/0.1	5/100	0.05/0.25	Fluid:<br>0.005/0.05<br>Sed: 0.05/0.05	yr

Supplementary Reference

1 Boyer, T. P. *et al.* World Ocean Database 2013. 209pp (2013).
